# Effects of External Stimulation on Psychedelic State Neurodynamics

**DOI:** 10.1021/acschemneuro.3c00289

**Published:** 2024-01-12

**Authors:** Pedro A. M. Mediano, Fernando E. Rosas, Christopher Timmermann, Leor Roseman, David J. Nutt, Amanda Feilding, Mendel Kaelen, Morten L. Kringelbach, Adam B. Barrett, Anil K. Seth, Suresh Muthukumaraswamy, Daniel Bor, Robin L. Carhart-Harris

**Affiliations:** † Department of Computing, Imperial College London, London SW7 2AZ, U.K.; ‡ Department of Psychology, University of Cambridge, Cambridge CB2 3EB, U.K.; § Department of Informatics, University of Sussex, Brighton BN1 9RH, U.K.; ∥ Centre for Psychedelic Research, Department of Brain Sciences, Imperial College London, London SW7 2AZ, U.K.; ⊥ Centre for Complexity Science, Imperial College London, London SW7 2AZ, U.K.; # Centre for Eudaimonia and Human Flourishing, University of Oxford, Oxford OX1 2JD, U.K.; ∇ The Beckley Foundation, Oxford OX3 9SY, U.K.; ○ Wavepaths, London WC2B 5AH, U.K.; ◆ Department of Psychiatry, University of Oxford, Oxford OX1 2JD, U.K.; ¶ Center for Music in the Brain, Department of Clinical Medicine, Aarhus University, Aarhus 8000, Denmark; ⋈ Sussex Center for Consciousness Science and Department of Informatics, University of Sussex, Brighton BN1 9RH, U.K.; ⧓ CIFAR Program on Brain, Mind, and Consciousness, Toronto M5G 1M1, Canada; ⧖ School of Pharmacy, Faculty of Medical and Health Sciences, The University of Auckland, Auckland 1023, New Zealand; ● Department of Psychology, Queen Mary University of London, London E1 4NS, U.K.; ¤ Psychedelics Division, Neuroscape, University of California San Francisco, San Francisco, California 94117-1080, United States

**Keywords:** complexity, psychedelics, neuroscience, consciousness

## Abstract

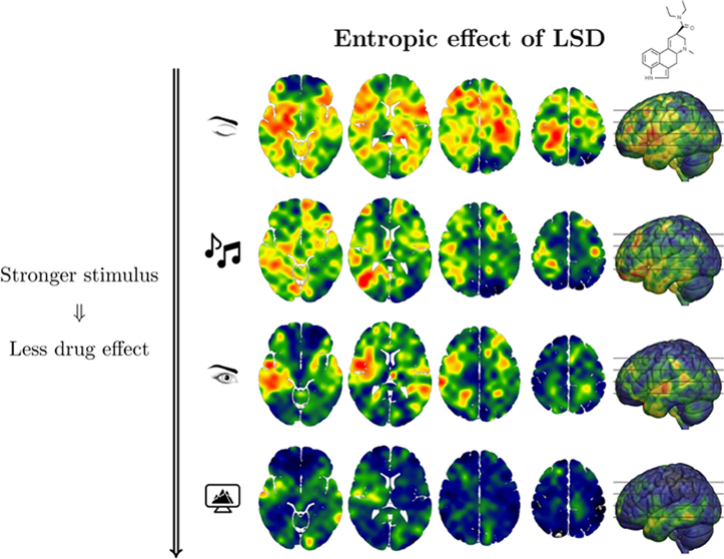

Recent findings have shown that psychedelics reliably enhance brain entropy (understood as neural signal diversity), and this effect has been associated with both acute and long-term psychological outcomes, such as personality changes. These findings are particularly intriguing, given that a decrease of brain entropy is a robust indicator of loss of consciousness (e.g., from wakefulness to sleep). However, little is known about how context impacts the entropy-enhancing effect of psychedelics, which carries important implications for how it can be exploited in, for example, psychedelic psychotherapy. This article investigates how brain entropy is modulated by stimulus manipulation during a psychedelic experience by studying participants under the effects of lysergic acid diethylamide (LSD) or placebo, either with gross state changes (eyes closed vs open) or different stimuli (no stimulus vs music vs video). Results show that while brain entropy increases with LSD under all of the experimental conditions, it exhibits the largest changes when subjects have their eyes closed. Furthermore, brain entropy changes are consistently associated with subjective ratings of the psychedelic experience, but this relationship is disrupted when participants are viewing a video—potentially due to a “competition” between external stimuli and endogenous LSD-induced imagery. Taken together, our findings provide strong quantitative evidence of the role of context in modulating neural dynamics during a psychedelic experience, underlining the importance of performing psychedelic psychotherapy in a suitable environment.

## Introduction

Psychedelic substances, such as lysergic acid diethylamide (LSD) and psilocybin, are known to induce profound changes in the subjects’ perception, cognition, and conscious experience. In addition to their role in ancestral spiritual and religious practices^1^ and their recreational use related to introspection and self-exploration,^2^ there is promising evidence that psychedelics can be used therapeutically to treat multiple mental health conditions.^3−6^ However, despite the increasingly available evidence of the neurochemical action of psychedelics at the neuronal and subneuronal level,^7,8^ the mechanisms associated with their therapeutic efficacy are not yet completely understood.

Some of the factors at play during psychedelic therapy can be related to the entropic brain hypothesis (EBH),^9,10^ a simple yet powerful theory which posits that the rich altered state of consciousness experienced under psychedelics depends on a parallel enriching effect on the dynamics of the spontaneous population-level neuronal activity.^1^ The hypothesis that increased brain entropy—as captured, e.g., by Lempel–Ziv (LZ) complexity^10^—corresponding to states of enriched experience has found empirical support in neuroimaging research on psychedelics^11,12^ as well as on other altered states, like meditation^13^ and states of “flow” associated with musical improvisation.^14^ Furthermore, the therapeutic mechanisms of psychedelics are thought to depend on their acute entropy-enhancing effect, potentially reflecting a window of opportunity (and plasticity) mediating therapeutic change.^15,16^ Conversely, states such as deep sleep, general anesthesia, and loss of consciousness have consistently shown reduced brain entropy.^17−19^

The effectiveness of psychedelic therapy is thought to depend not only on direct neuropharmacological action but also on contextual factors—commonly referred to as *set and setting*. These include the subject’s mood, expectations, and broader psychological condition (set) prior to the “trip”, together with the sensorial, social, and cultural environment (setting) in which the drug is taken. For example, there is direct evidence that specific music choices may either enhance or impede therapeutic outcomes^20^ and that the social setting in which a psychedelic experience takes place facilitates positive long-term effects.^21^ To the best of our knowledge, this paper presents the first quantitative analysis showing that this effect of the setting can be detected from physiological measurements directly.

Despite its presumed importance, to our knowledge, no previous study has systematically assessed the influence of set and setting on brain activity and subjective experience during a psychedelic experience. This lack of relevant research, combined with the fact that psychedelic therapy is almost exclusively carried out with music listening and eyes closed, exposes a knowledge gap that compromises key assumptions of current psychedelic therapy practice. Here, we provide a first step toward bridging this gap, presenting a systematic investigation of how different environmental conditions can modulate changes in brain entropy elicited by psychedelics in healthy subjects. This work provides a proof of principle that paves the way for future studies with clinical cohorts.

## Materials and Methods

### Data Collection and Preprocessing

We used the data presented by Carhart-Harris et al.,^22^ together with previously unpublished data from the same experiment. Twenty subjects participated in the study by attending two experimental sessions: one in which they received intravenous (i.v.) saline (placebo) and one in which they received i.v. LSD (75 μg). The order of the sessions was randomized, separated by 2 weeks, and participants were blind to the order (i.e., a single blind design). Whole-brain magnetoencephalography (MEG) data were collected under four conditions: resting state with eyes closed, listening to instrumental ambient music with eyes closed, resting state with eyes open (focusing on a “fixation dot”), and watching a silent nature documentary video—henceforth referred to as *closed, music, open*, and *video*. The music tracks were taken from the album “Eleusian Lullaby” by Alio Die, and the video was composed of segments of the “Frozen Planet” documentary series produced by the BBC. More information about the experimental design can be found in the original study.^22^

**Figure 1:**
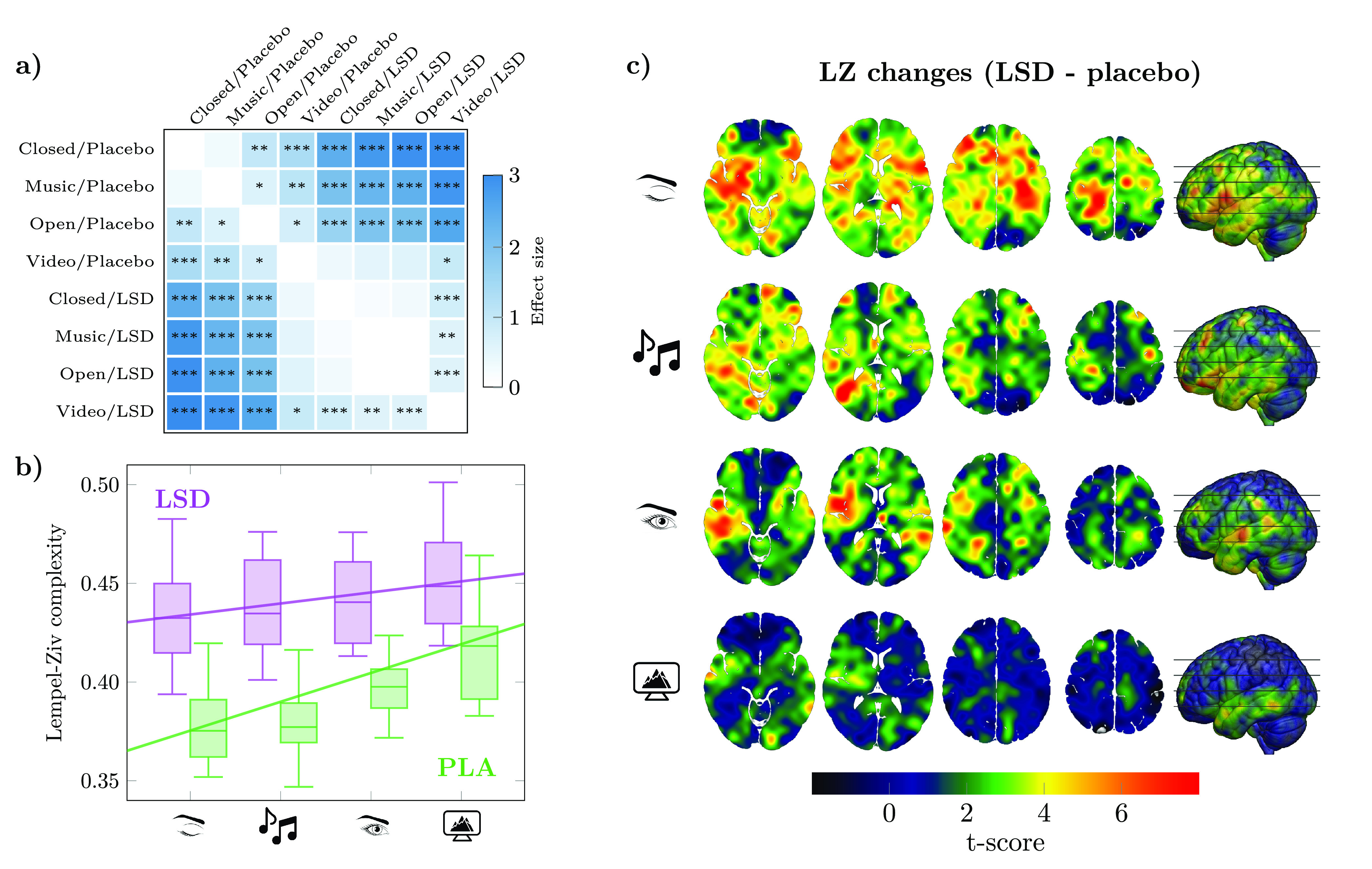
Stronger external stimulation increases baseline entropy and reduces the drug effect. (a) Differences in average LZ, as measured by posthoc *t* tests and effect sizes (Cohen’s d), increase with stimulus and the drug (*:*p* < 0.05,**: *p* < 0.01,***: *p* < 0.001). (b) However, stronger external stimulation (i.e., with higher baseline LZ) *reduces* the differential effect of LSD on brain entropy vs placebo. Linear mixed-effects models fitted with LZ complexity as the outcome show a significant negative drug × condition interaction (*p* < 0.01; see Supporting Table S1). (c) T-scores for the effect of the drug under all four experimental conditions. In agreement with the LME models, the effect of the drug on increasing LZ substantially diminishes with eyes open or under external stimuli.

MEG data were collected with a 271-gradiometer CTF MEG scan. In addition, structural MRI scans of every subject were obtained for later intersubject coregistration. Three subjects could not complete all stages of recording or had excessive movement artifacts and were removed from the analysis altogether. All preprocessing steps were performed using the FieldTrip toolbox.^23^ First, artifacts were removed by visual inspection, and muscle and line noise effects were removed using ICA. Then, we applied a second-order low-pass Butterworth filter at 100 Hz and split the data into 2 s epochs for subsequent analysis. For source reconstruction, we used the centroids of the AAL-90 atlas.^24^ The positions of these centroids were nonlinearly inverse-warped to subject-specific grids using the subjects’ structural MRI scans, and source time series (a.k.a. *virtual sensors*) were estimated with a regularized LCMV beamformer. We calculated Lempel–Ziv complexity (LZ; see below) on these locations and finally mapped them back onto the standard template for statistical analysis and visualization. In addition, for the visualization in Figure 1c, we computed LZ in sources reconstructed in a uniform 10 mm three-dimensional (3D) grid.

In addition to MEG and MRI measurements, visual analogue scale (VAS) subjective ratings were collected at the end of each session. The questionnaires were designed to capture central features of the subjective effects of LSD. They included assessments of the intensity of the experience, emotional arousal, ego dissolution, positive mood, and simple and complex internal visual imagery. The imagery items were rated only for the eyes-closed conditions.

### Lempel–Ziv Complexity

The main tool of analysis used in this study is Lempel–Ziv complexity (referred to as LZ), which estimates how diverse the patterns exhibited by a given signal are.^25^ The method was introduced by Lempel and Ziv to study the statistics of binary sequences^25^ and was later extended^26,27^ to become the basis of the well-known “zip” compression algorithm. This algorithm has been used to study the diversity of patterns in EEG activity for more than 20 years, with some early studies focusing on epilepsy^28^ and depth of anesthesia.^29^

LZ is calculated in two steps. First, the value of a given signal *X* of length *T* is binarized, calculating its mean value and turning each data point above it to “1”s and each point below it to “0”s. Then, the resulting binary sequence is scanned sequentially looking for distinct structures or “patterns.” Finally, signal complexity is determined by the number of patterns found, denoted by *C*_LZ_(*X*). Regular signals can be characterized by a small number of patterns and hence have low *C*_LZ_, while irregular signals contain many different patterns and hence have high *C*_LZ_.

Following the reasoning above, the LZ method identifies signal complexity with *richness of content*(30)—a signal is considered complex if it is not possible to provide a brief (i.e., compressed) representation of it. Accordingly, a popular way of understanding LZ is as a proxy for estimating Kolmogorov complexity, the length of the shortest computer program that can reproduce a given pattern.^31^ However, we (and others) argue that this view is brittle in theory and of limited use in practice.^32^ A simpler and more direct interpretation of LZ is to focus on the quantity
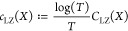
which is an efficient estimator of the entropy rate of *X*.^33^ The entropy rate measures how many bits of innovation are introduced by each new data sample^34^ and is related with how hard it is to predict the next value of a sequence.^2^ This makes this normalized LZ, *c*_LZ_, a principled, data-efficient estimator of the diversity of the underlying neural process. For simplicity, the rest of the article refers to *c*_LZ_ generically as LZ.

In terms of algorithm, we follow the original procedure presented by Lempel and Ziv in 1976^25^—commonly known as LZ76—computed with the simplified algorithm described by Kaspar and Schuster.^36^ We note that although other versions of the LZ algorithm can also be employed to estimate the entropy rate (e.g., the common dictionary-based implementation^26,27^), their computation time and convergence are slower than LZ76, making the latter a better choice for our experiments. Unlike in previous studies, we do not apply a Hilbert transform and instead apply the LZ procedure to the source-reconstructed, broadband signal. While there are certain interpretability advantages to using a Hilbert transform (for example, signal can be interpreted as the amplitude of an underlying neural oscillation), the Hilbert transform cannot be meaningfully applied to broadband signals, and prefiltering the data would add further (undesired) degrees of freedom to our analysis. In practice, however, LZ is a remarkably robust measure and the same qualitative results hold under different preprocessing techniques. See references (11,17,37) for further discussion.

### Brain Regions of Interest

For the neural-psychometric correlation analysis, as shown in Figure 3 onward, we calculated the average LZ of several brain regions of interest (ROIs), each of them composed of a number of subregions represented in the AAL-90 atlas.^24^ For each subject, the mean LZ value of each ROI was obtained by averaging the LZ values of the source-reconstructed activity at the centroid of each subregion.

For all of the analyses, the following ROIs were considered: two sensory areas, two related to the DMN, one related to interoception, and one to emotion. Specifically, the considered ROIs and their corresponding AAL-90 subregions are

- auditory: left and right Heschl areas
- visual: left and right calcarine, bilateral lingual, cuneus, inferior, middle, and superior occipital
- amygdala: both left and right
- insula: both left and right
- mPFC: left and right medial superior frontal gyrus; and
- posterior DMN: bilateral posterior and median cingulate gyrus, middle temporal gyrus, and angular gyrus.

### Statistical Modeling

To explore the effect of external conditions in detail, disentangling the effect of stimuli versus an effect *beyond* merely opening one’s eyes, we encoded the experimental condition in two binary variables: eyes-open (true for the *open* and *video* conditions, false otherwise) and stimulus (true for the *music* and *video* conditions, false otherwise).

The paper considers various linear mixed-effects (LME) models, in most cases with a measure of interest (VAS ratings or LZ complexity) as target; drug, stimulus, and eyes-open as fixed effects; and subject identity as a random effect. When constructing a model, all possible pairwise interactions were considered; then, model selection was performed using the Bayesian information criterion (BIC). All of the reported models corresponded to the one selected by the BIC. All models were estimated via restricted maximum likelihood, using the open-source packages lme4 v.1.1–21(38) and lmerTest v.3.1–1(39) on R v.3.6.0.

Finally, we used these LME models to perform conditional predictive analyses, according to the following procedure. Consider the case of studying the conditional predictive power of LZ in a given ROI *R*_1_ with respect to a particular subjective report *V*. We say that the predictive power from *R*_1_ to *V* is statistically mediated by another ROI *R*_2_ if the two conditions are satisfied. First, LZ in both *R*_1_ and *R*_2_ is significantly correlated with *V* according to their respective BIC-optimal (as per the previous paragraph) LME model—i.e., the FDR-corrected *p*-value of their estimated regression coefficients is below 0.05. Second, when calculating a BIC-optimal LME model with *V* as target and LZ of both *R*_1_ and *R*_2_ as predictors (plus controlling variables), the estimate of the effect of *R*_1_ loses significance—i.e., its non-FDR-corrected *p*-value goes above 0.1. Using the outcomes of these analyses, we build diagrams of the predictive ability of various variables (as the ones shown in Figure 5), in which we add an arrow from *R*_1_ to *R*_2_ if *R*_2_ mediates the relationship between *R*_1_ and *V* or an arrow from *R*_1_ to *V* if there is no other variable that mediates their relationship.

## Results

### Increased LZ under External Stimulation

Studying the whole-brain average LZ from the placebo sessions showed that external stimuli yield significant differences in LZ (Kruskal–Wallis test, *p* < 0.001). Posthoc *t* tests, as shown in Figure 1a, revealed that richer stimuli induce consistent significant increases across conditions, with large effect sizes (Cohen’s *d*).

To disentangle the effect of the stimuli over the effect of eye opening, a linear mixed-effects (LME) model was constructed using the presence of stimulus and eye opening as predictor variables and subject identity as a random effect (see Materials and Methods Section). This model showed significant positive effects of both stimulus (β = 0.013, SE = 0.005, *p* = 0.017) and eye opening (β = 0.025, SE = 0.005, *p* < 0.001). The statistical significance of both effects suggests that the measured LZ cannot be explained merely by the presence or absence of visual stimuli and must be related to the structure of such stimuli (either music or video). Nonetheless, it is noteworthy that the simple act of opening one’s eyes has an especially marked (augmenting) effect on brain entropy.

### Stronger External Stimulus Weakens the Drug Effect

To study the effect of LSD on the whole-brain average LZ, we constructed LME models similar to those in the previous section and added the drug as a fixed effect. This analysis shows a dramatic increase in LZ under the effects of LSD (β = 0.047, SE = 0.005, *p* < 0.001), much larger than that associated with eye opening or stimulus (Figure 1b). Posthoc analyses showed that the effect of the drug is substantial in all stimulus conditions (Figure 1a).

Crucially, the LME model revealed a significant interaction between the drug and eye opening as predictors of LZ (β = −0.016, SE = 0.006, *p* = 0.011). Importantly, this interaction effect was negative—i.e., the increased external stimulation *reduced* the effect of the drug. Alternatively, this can be interpreted as the drug reducing the effect of external stimulation on brain entropy—which, either way, points toward a “competition” between endogenous, drug-induced and exogenous, stimulus-induced effects on neural dynamics.^40^ This negative interaction was confirmed by ordering the four experimental conditions with integer values from 1 to 4 (Figure 1b) and with multiple statistical hypothesis tests (e.g., 2-way ANOVA; see Supporting Table S2). Furthermore, we confirmed that the results still hold with stricter filters (e.g., a low-pass filter at 30 Hz on the MEG signals) and when controlling for order effects between the stimulus and nonstimulus sessions (see Supporting Tables S3 and S4). Both the effect of the drug and its interaction with external conditions are spatially widespread (Figure 1c).

As a further confirmatory analysis, we computed the spectral power in the α (8–13 Hz) frequency band since α suppression is a known correlate of the psychedelic state.^41^ As expected, LME modeling revealed a strong decrease in α power driven by LSD (β = −6.31 × 10^–43^, SE = 6.81 × 10^–44^, *p* < 0.001) as well as an interaction effect of the opposite sign between the drug and eye opening (β = 3.57 × 10^–43^, SE = 9.56 × 10^–44^, *p* < 0.001; see Supporting Figures S3 and S4 and Table S12). This supports the same conclusion as the LZ results that a stronger external stimulus weakens the drug’s effect. Importantly, however, although α power is a strong correlate of the psychedelic state, as we show in the rest of the Results, it is far less predictive of subjective results than LZ.

### Setting Modulates Subjective Ratings and their Relationships

**Figure 2:**
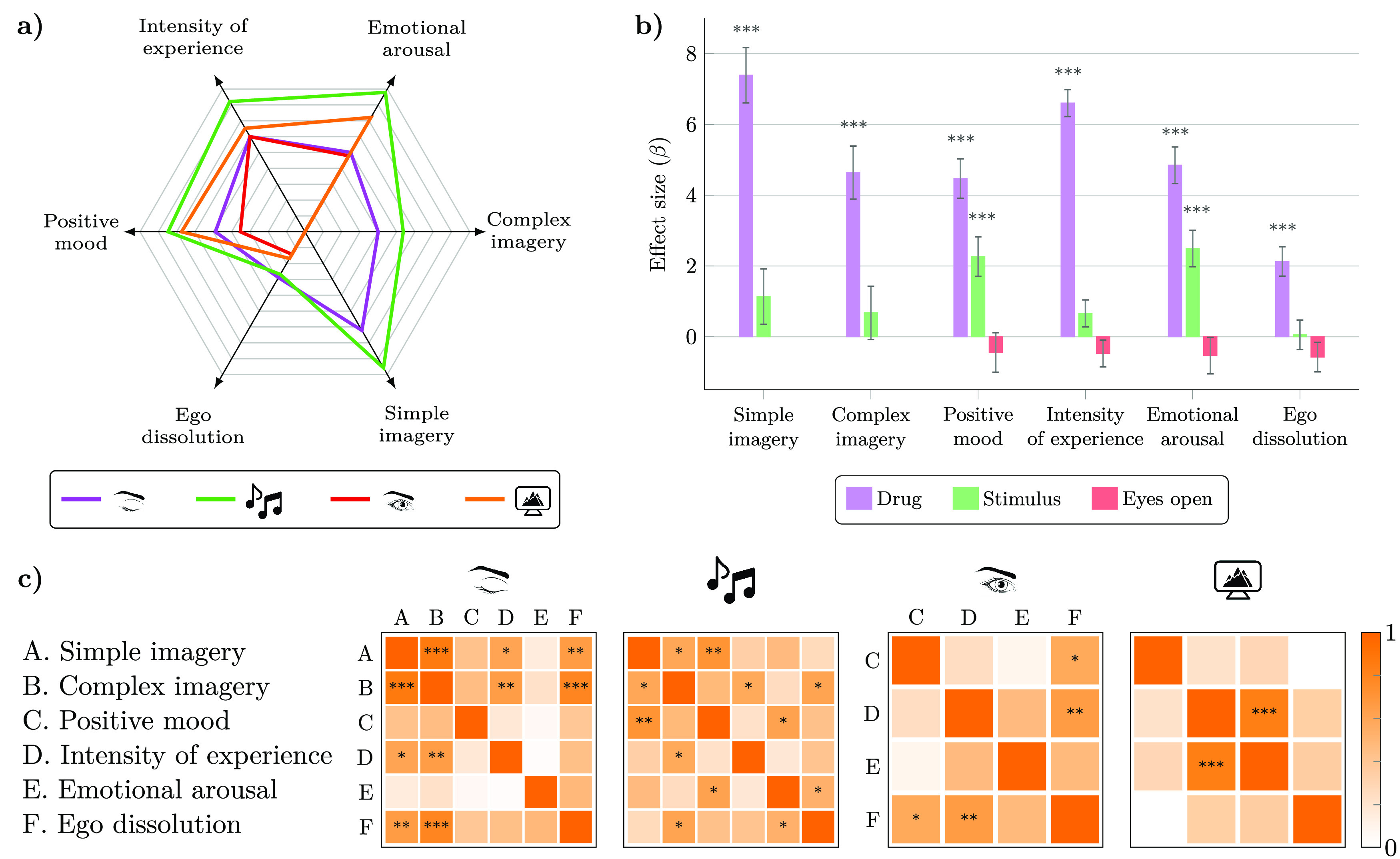
Setting affects participants’ subjective reports of their psychedelic experiences. (a) Average increases in VAS ratings between LSD and placebo show a varied profile across experimental conditions, suggesting that setting modulates participants’ rating of their own experience. Simple and complex imagery data were not collected under the eyes-open and video conditions. (b) Effect sizes obtained from LME modeling confirm a strong effect from the drug in all items, as well as smaller and more specific effects from the stimulus. (c) Between-subjects correlation matrices between experience reports (*: *p* < 0.05,**: *p* < 0.01,***: *p* < 0.001).

The effects of LSD on VAS ratings varied widely between the conditions (Figure 2a). A quantitative LME analysis showed the effect of the drug to be much larger than that of the stimulus or eye opening on all of the VAS measures (Figure 2b). Additionally, stimulus effects tended to be more specific than drug effects, reaching statistical significance only for positive mood and emotional arousal—in line with previous findings that carefully selected stimuli (e.g., music) can boost the affective state of subjects undergoing psychedelic psychotherapy.^20,42,43^ It is worth noting that these two are the least psychedelic-specific items.

Differences in setting not only affected the subjects’ VAS ratings but also the relationship between the ratings themselves (Figure 2c). For example, when resting with eyes closed, subjects tended to rate the intensity of their experience in agreement with the vividness of their simple and complex imagery—but, when watching a video, the intensity was more strongly correlated with emotional arousal. These findings show that what subjects consider their intensity of experience can dramatically vary across various dimensions,^44^ confirming the assumption that the subjective quality and general intensity of a psychedelic experience strongly depends on the environmental conditions (or setting) in which it takes place.

### Neural-Psychometric Correlations Can Be Disrupted by External Stimuli

A major aim of psychedelic neuroimaging is to discover specific relationships between brain activity and subjective experience. Examples include mappings between specific neural dynamics and ratings of ego dissolution^45^ or other specific aspects of experience such as its visual quality.^11^ However, given that—as we show here—setting interacts with neural dynamics, it is natural to ask whether it also affects the relationship between phenomenology and its neural correlates.

To address this question, we analyzed the relationship between LZ and VAS changes induced by LSD in each one of the four experimental conditions. Between-subjects Pearson correlation coefficients were calculated between changes in VAS ratings and LZ measured in different regions of interest (ROIs). Motivated by the nature of the study and known brain effects of LSD,^22,45^ we focused on areas associated with sensory processing (visual and auditory), interoception (insula), emotional processing (amygdala), and self-monitoring (mPFC and posterior DMN; see Materials and Methods Section for details).

**Figure 3:**
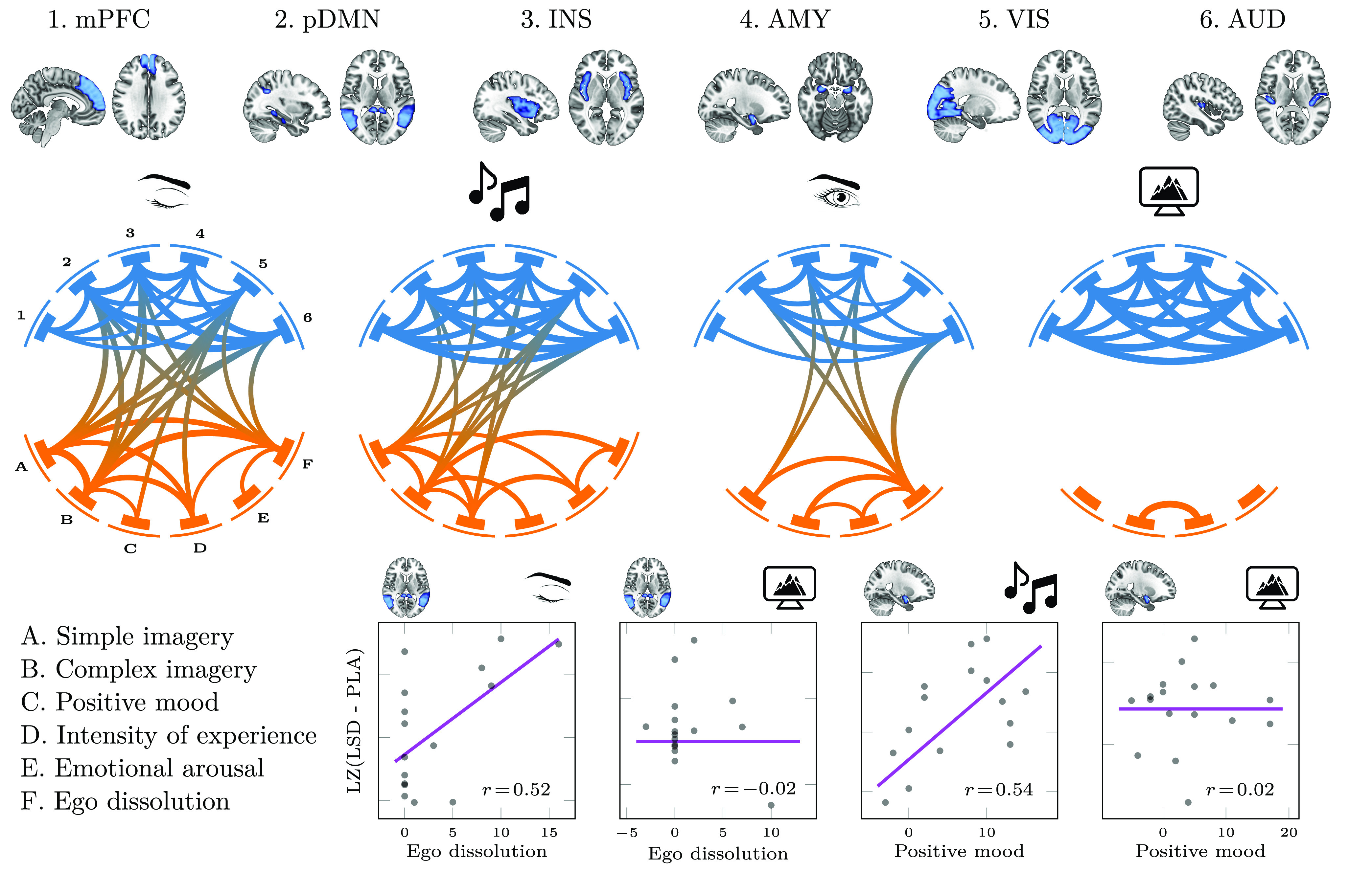
External stimulation alters the relationship between the psychometric and neural effects of LSD. The network representation of correlation matrices between brain entropy in six regions of interest (numbered 1–6, top) and subjective experience ratings (labeled A–F, bottom left) in all four experimental conditions is shown. As external stimulation is increased, there is a large decrease in the correlation between subjective ratings and entropy, but an increase in the correlation in entropy between different brain regions (see Supporting Figure S1 and Tables S5 and S6). The bottom right panels show example correlations between ego dissolution and posterior DMN entropy (two left panels) and positive mood and amygdala entropy (two right panels). In both cases, the correlation is strong and significant with eyes closed, but vanishes when subjects watch a video.

Analyses revealed multiple significant relationships between subjective ratings and LZ changes during the closed, music, and open conditions (Figure 3). For example, we observed significant (*p* < 0.05, FDR-corrected) positive correlations between ego dissolution and DMN, positive mood and amygdala, and simple and complex imagery and visual and auditory ROIs, all in the eyes-closed condition—supporting the suitability of the eyes-closed resting condition for assessing the neural correlates of these experiences. Strikingly, all of the observed neural-psychometric correlations vanished when subjects watched a video, with none exceeding an absolute value of |*r*| > 1/10. This observation was verified by building a multivariate regression model, using the correlation coefficients between VAS and LZ changes as target variables and stimuli and eye opening as predictors. Results showed that neither stimuli (*p* = 0.17) nor eyes-open (*p* = 0.13) had significant effects by themselves, but their interaction was strongly associated with smaller VAS–LZ correlation values (β = −0.21, SE = 0.08, *p* = 0.006; see Supporting Table S5).

As a complementary analysis, we also studied how the four environmental conditions affect the relationship among the LSD-induced LZ changes across different ROIs. To do this, we evaluated the Pearson correlation coefficient between the LZ changes measured in the various ROIs across subjects. It was observed that the correlation between ROIs is substantially increased when subjects perceive an external stimulus (either music or video; see Supporting Table S6), which could be indicative of a form of “complexity matching”,^46^ in which neural dynamics are entrained by the external stimulus, obscuring the relationship between neurodynamics and subjective experience. This observation was also verified via multivariate regression modeling, this time using ROI–ROI correlation values as target. In this case, eye opening was associated with smaller correlation values (β = −0.10, SE = 0.04, *p* = 0.011), while stimuli (β = 0.15, SE = 0.04, *p* < 0.001) and the interaction between stimuli and eyes-open (β = 0.18, SE = 0.05, *p* = 0.001) were both associated with significantly larger correlation values (see Supporting Table S6). These results were also controlled for the effect of ordering between experimental conditions (see Supporting Figure S2 and Tables S9 and S10). These findings suggest that the increased within-brain correlation driven by external stimulation may obfuscate potential correlations between entropy and individual VAS ratings—which are most apparent, e.g., in the eyes-closed condition.

### Conditional Predictive Analyses of Subjective Reports

Finally, we analyzed the relationship between changes in LZ and behavioral reports as they were exposed to the different experimental conditions. For this, we constructed LME models using VAS ratings as target; average LZ, eye opening, and stimulus as fixed effects; and subject identity as a random effect (see Materials and Methods Section).

**Figure 4:**
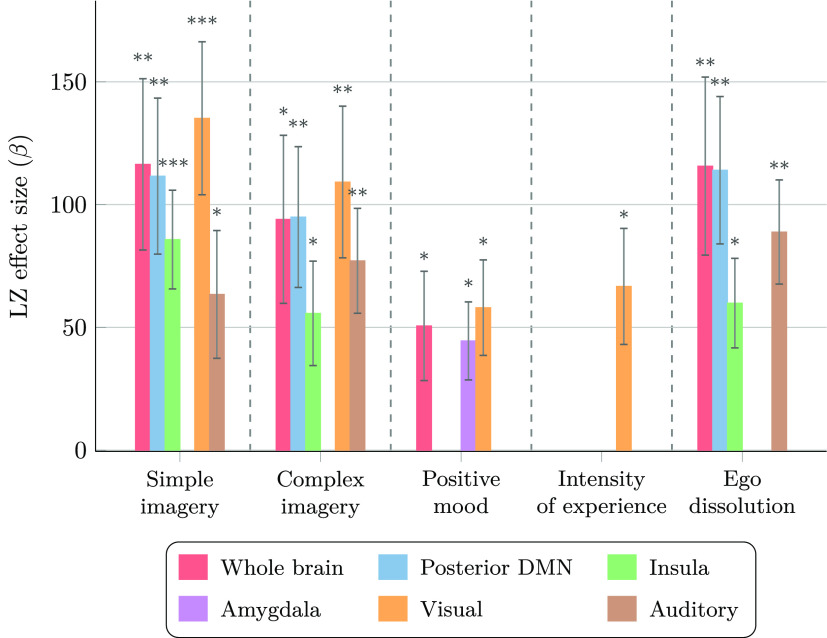
Changes in brain entropy predict changes in subjective reports. Estimates, standard error, and FDR-corrected statistical significance (*: *p* < 0.05,**:*p* < 0.01,***: *p* < 0.001) of the effect of the LZ differences (LSD-PLA) for predicting VAS differences (LSD-PLA), obtained from LME models calculated over the four conditions.

These models revealed multiple associations between brain entropy and subjective reports (Figure 4), including some widespread correlations with LZ averaged across the whole brain (most strongly with ego dissolution and simple imagery), as well as several more specific correlations (e.g., between positive mood and amygdala, and between simple imagery and visual regions). In contrast, stimulus and eye opening show small effect sizes in all models and strong negative interactions with LZ (see Supporting Table S11), suggesting that the relationship between LZ and VAS is broken when stimuli are present, in line with the results shown in Figure 3. We also analyzed similar models using α power instead of LZ, and found that α power (despite changing drastically between conditions) is a poor predictor of subjective reports, with only three ROIs showing significant correlations with two VAS items after FDR correction (see Supporting Figure S5). For comparison with LZ, all six ROIs show significant correlations with one or more VAS items, and all VAS items are predicted by one or more ROIs.

**Figure 5:**
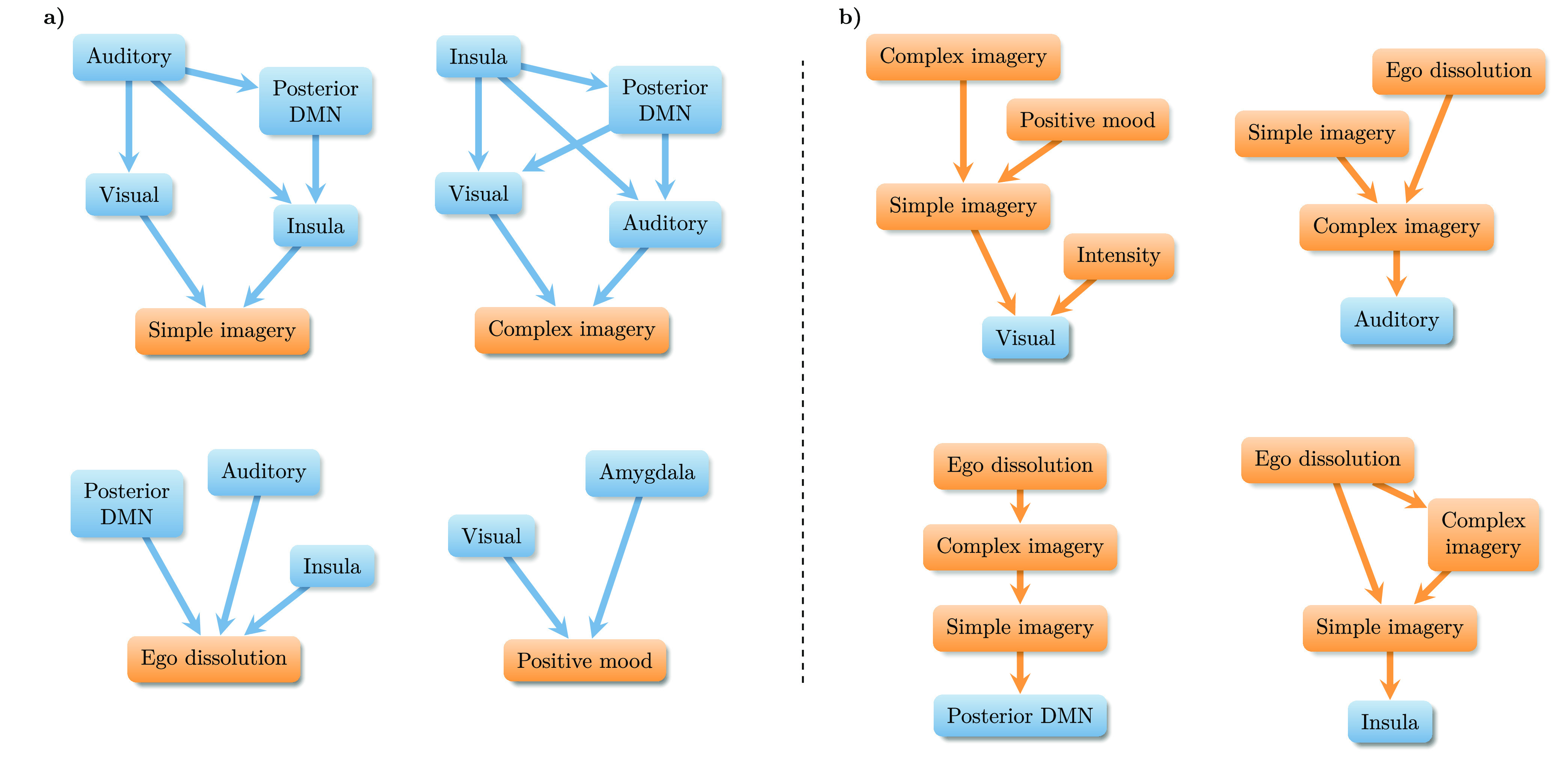
Statistical structure of brain entropy and subjective ratings data. Networks represent the conditional prediction diagrams (see Materials and Methods Section) in which node *i* is connected to node *j* if *j* “mediates” the statistical predictive information that *i* has about a target variable (bottom node in each network). Conditional predictive analysis (a) from brain entropy to subjective experience reports, and (b) from subjective reports to brain entropy.

To explore the correlations between behavioral ratings and LZ in various ROIs in more detail, we performed a conditional predictive power analysis (see Materials and Methods Section). This method allows us to build a directed network representing the predictive ability of the various ROIs with respect to a given VAS item, such that a ROI *R*_1_ is connected to a VAS item *V* via another ROI *R*_2_ if, once the entropy change in *R*_2_ is known, there is no further benefit in knowing the entropy change in *R*_1_ for improving the prediction of the change in *V* (Figure 5a). Results show that, in general, “low-level” regions (i.e., closer to the sensory periphery, like visual areas) tend to “mediate” the associations between subjective reports and high-level regions (like the DMN). For example, visual and auditory areas mediate the predictive information that the pDMN and insula have about reported complex imagery.^3^ Put simply, once the change in entropy in the auditory and visual regions is known, knowing the change in entropy in the pDMN provides no extra information about the change in the reported complex imagery. A notable exception, however, is ego dissolution, for which the pDMN, auditory, and insula all provide unmediated complementary information—in line with previous studies linking self-related processing and the DMN.^22^

We also performed a reciprocal analysis to assess the conditional predictive power of the various VAS items using LZ as the target (Figure 5b). Results show that, across brain regions and VAS items, the predictive power of more abstract VAS scores (e.g., ego dissolution, positive mood) tends to be mediated by less abstract ones (e.g., simple and complex imagery). For example, changes in ego dissolution scores become irrelevant for predicting LZ in auditory areas once one knows the corresponding change in complex imagery. One interpretation of these analyses is that brain entropy, as currently measured with LZ, may most faithfully reflect “low-level” aspects of the brain–mind relation (see the Discussion Section).

## Discussion

The present study’s findings provide strong quantitative evidence on how environmental conditions can have a substantial influence on both subjective experience and neural dynamics during a psychedelic experience. Importantly, the entropy-enhancing effects of LSD were less marked when participants opened their eyes or perceived external stimuli—such as music or video. Furthermore, the differences in brain entropy observed in various regions of the brain were found to be associated with behavioral reports about the subjects’ perception, emotion, and self-related processing—but the relationship between brain entropy and subjective reports collapsed in the video-watching condition.

### LZ as a Robust Correlate of Subjective Experience

The increase in brain entropy—seen via LZ—is known to be a robust M/EEG biomarker associated with the psychedelic state^11,12^ and, indeed, conscious states, more generally.^17−19,47^ In addition to replicating this effect on new data, we also observed other known effects of serotonergic psychedelics, including pronounced spectral power changes (in particular, the LSD-induced α suppression^41^). Interestingly, the relationship between changes in these other metrics (like α power) and subjective ratings was substantially weaker than that of LZ (see Supporting Figure S5), suggesting that LZ is a particularly well-suited marker of psychedelic subjective experience.^4^

Notably, subjects under LSD watching a video had the highest absolute brain entropy but did not give maximal subjective ratings in any of the psychometric items. Furthermore, while a profound subjective experience such as ego dissolution was found to correlate with LZ changes, this effect was found most prominently in the eyes-closed condition, and its predictive power was mediated by reported (simple and complex) visual imagery. These results suggest that LZ may reflect a nuanced combination of both endogenous and exogenous factors.

We propose two alternative interpretations of these findings. On the one hand, it could be that LZ most faithfully indexes brain activity associated with low-level sensory processing. On the other hand, it could be that LZ shows strong associations with high-level cognitive processing or subjective phenomena (such as ego dissolution) in the eyes-closed conditions because that relationship becomes more specific in the absence of the strong sensory “driving” effects present in the eyes-open conditions—especially video. Future studies might distinguish between these hypotheses by exploring the reliability of relationships between LZ and various subjective phenomena, including ego dissolution, perceptual complexity, and alertness, involving different pharmacological agents (e.g., psychedelics and stimulants), dosages, and stimuli.

### Toward a Refinement of the Entropic Brain Hypothesis

A deeper understanding of the functional relevance of brain entropy will help us better understand how such measures can be refined in order to shed clearer light on their relationship with the reported phenomenology. The results presented in this paper, while grounded in and motivated by the EBH, also highlight some important qualifiers of it. Since brain entropy measures such as LZ depend only on the dynamics of individual loci (e.g., individual time series corresponding to single sources or sensors), they may only indirectly reflect the richer scope of brain dynamics, network, and connectivity properties—although it is worth noting that the LSD-induced entropy increases at the single-source level have been related to specific network properties of the human connectome.^49^

One potential way forward for the EBH may be to consider the entropy of network dynamics and other high-order brain features rather than merely the entropy of individual sources. For example, examining increases in entropy at the level of emergent whole-brain states may prove particularly fruitful.^50^ We see this as part of a broader move toward multidimensional descriptions of brain activity, transcending the “one-size-fits-all” scalar measures—including more complicated unidimensional ones like integrated information.^51,52^ In line with recent theoretical proposals^53^ and experimental findings,^12^ a range of metrics may be necessary to provide a more complete, multidimensional representation of brain states. However, we also acknowledge that increasing model complexity can complicate interpretability and affect statistical power and thus is only justified when it yields substantial improvement in explanatory power and is driven by reliable hypotheses.

### Implications for Psychedelic Psychotherapy

These findings can be regarded as neurobiological evidence for the importance of environmental context,^54^ or ‘setting’, to the quality of psychedelic experiences—a matter of particular relevance to psychedelic therapy. In particular, the present findings support the principle that having one’s eyes closed during a psychedelic experience may enhance the differential entropic effect of the drug,^3^ which is consistent with approaches fostering eyes-closed, introspective experiences during psychedelic therapy, as they may lead to beneficial therapeutic outcomes.^55^ In addition, our results suggest a differential effect between sensory modalities (visual versus auditory) on brain dynamics and subjective experience with visual stimulation reducing the measured relationship between neural entropic changes and subjective reports. Together, these findings support the choice of music—in contrast to visual stimulation—to modulate and support psychedelic therapy.^20,56,57^

It remains possible that environments or stimuli different from the ones considered in this study could potentially lead to different results. Additionally, there are a number of phenomena relevant to the psychedelic experience for which having eyes open may be more conducive (e.g., feelings of communitas or acute connection with nature^58^), which cannot be assessed within the current experimental design. Furthermore, the observed disruption between psychological phenomena and brain dynamics was only assessed via LZ applied to MEG data and might not be true for other neural signatures.

Importantly, this study reveals that the effects of contextual elements on brain dynamics can be effectively tracked via current neuroimaging techniques. Our results establish LZ as a marker that is sensitive to the interaction between the drug and context, which opens the door to future studies that may assess the effect of contextual elements on the brain during psychedelic therapy. This study therefore serves as a proof-of-concept translational investigation in healthy subjects, setting a precedent for future studies in clinical populations. Accompanying extensions into clinical populations, future work is also needed to further clarify how interactions between the drug and context manifest on a psychological and neurobiological level and how they can be harnessed for best therapeutic outcomes.

### Ethics Statement

This study was approved by the National Research Ethics Service Committee London-West London and was conducted in accordance with the revised declaration of Helsinki (2000), the International Committee on Harmonization Good Clinical Practice guidelines, and the National Health Service Research Governance Framework. Imperial College, London sponsored the research, which was conducted under a Home Office license for research with Schedule 1 drugs.

## Supporting Information Available

Methodology used for the additional controls and discussions on the supporting results; numerical results of the statistical models used throughout the analyses (PDF)

## Footnotes

1 Entropy is understood here not as a thermodynamic but as an informational property, measuring the complexity of neural dynamics and the diversity of their configuration repertoire (see Materials and Methods Section).

2 In effect, the mean entropy rate divided by two approximates the probability of making an error with the best informed guess about the next sample.^35^

3 Although, note that the role of auditory regions and insula is reversed for simple and complex imagery, respectively.

4 The relation between LZ and spectral changes can be disentangled with more elaborate statistical methods,^48^ although this analysis is beyond the scope of this paper.
